# Decreased Plasma Levels of Angiotensin-Converting Enzyme Among Patients With Bipolar Disorder

**DOI:** 10.3389/fnins.2021.617888

**Published:** 2021-02-11

**Authors:** Marsal Sanches, Gabriela D. Colpo, Valeria A. Cuellar, Taya Bockmann, Deevakar Rogith, Jair C. Soares, Antonio L. Teixeira

**Affiliations:** ^1^Department of Psychiatry and Behavioral Sciences, UT Center of Excellence on Mood Disorders, The University of Texas Health Science Center at Houston, Houston, TX, United States; ^2^Neuropsychiatry Program, Department of Psychiatry and Behavioral Sciences, McGovern Medical School, The University of Texas Health Science Center at Houston, Houston, TX, United States; ^3^School of Biomedical Informatics, The University of Texas Health Science Center at Houston (UTHealth), Houston, TX, United States

**Keywords:** bipolar disorder, mood disorders, depression, renin-angiotensin system, angiotensin II, angiotensin converting enzyme

## Abstract

**Background:**

Dysfunctions in the renin-angiotensin system (RAS) seem to be involved in the pathophysiology of several mental illness, including schizophrenia and mood disorders. We carried out a cross-sectional study assessing the levels of RAS-related molecules among bipolar disorder (BD) patients compared to healthy controls.

**Methods:**

our sample consisted of 30 outpatients with BD type 1 (10 males, 20 females, age = 35.53 ± 10.59 years, 14 euthymic, 16 experiencing mood episodes) and 30 healthy controls (10 males, 20 females, age = 34.83 ± 11.49 years). Plasma levels of angiotensin-converting enzyme (ACE), angiotensin-converting enzyme 2 (ACE2), angiotensin-II (Ang II), and angiotensin (1–7) [Ang-(1–7)] were determined by ELISA.

**Results:**

BD patients experiencing ongoing mood episodes had significantly lower ACE levels compared to controls (median: 459.00 vs. 514.10, *p* < 0.05). There was no association between the levels of these biomarkers and clinical parameters.

**Conclusion:**

Our findings support the involvement of RAS dysfunction in the pathophysiology of BD. Considering the potential therapeutic implications linked to a better understanding of the role of RAS dysfunction in BD, studies allowing a better characterization of RAS-related molecules level and activity across different mood states are of high interest.

## Introduction

Bipolar disorder (BD) is a chronic and potentially severe mental illness that affects 2–5% of the American population (Merikangas et al., 2007). It is implicated in a high rate of morbidity and important functional impact (Kessler et al., 2006).

The pathophysiology of BD has not yet been completed elucidated but seems to involve multiple dimensions, including dysfunctions in the brain circuits associated with the processing of emotions, neurodevelopmental disruptions, and systemic processes (Brambilla et al., 2008; Sanches et al., 2008; Goldstein et al., 2009; Barbosa et al., 2014; Sanches and Soares, 2016). More recently, it has been hypothesized that dysfunctions in the renin-angiotensin system (RAS) might play a role in the pathophysiology of BD (de Góis Queiroz et al., 2013; Mohite et al., 2020).

Systemically, the RAS plays a crucial role in blood pressure regulation and in the maintenance of homeostasis (Ito et al., 1995). The existence of a brain RAS is well-established, and angiotensin-II receptors can be found in different areas of the brain, including amygdala, hippocampus, and prefrontal cortex (Reinecke et al., 2018). Angiotensin II (Ang II) is produced through the conversion of Angiotensin I by the action of the angiotensin-converting enzyme (ACE). Abnormal levels of ACE in the cerebrospinal fluid (CSF) and plasma of patients with schizophrenia (Wahlbeck et al., 1998; Baskan et al., 2010; Mohite et al., 2018), have been previously reported (Mohite et al., 2018). These findings seem to be of particular importance, in light of the possible role of the RAS in regulating the inflammatory response and the large amount of evidence supporting the involvement of inflammation in the pathogenesis of different mental illnesses, such as major depressive disorder (MDD), schizophrenia, and BD (Bauer and Teixeira, 2019). Furthermore, in Alzheimer’s disease, decreased ACE levels in plasma and CSF have been previously described (Jochemsen et al., 2014), and it has been hypothesized that these decreases are directly related to the accumulation of amyloid plaques and, ultimately, neuronal damage (Rocha et al., 2018b).

Specifically with respect to mood disorders, retrospective data point to lower levels of depressive and anxious symptoms among hypertensive patients receiving ACE inhibitors, as well as to lower rates of antidepressant use among patients with hypertension or diabetic nephropathy treated with ACE inhibitors or angiotensin receptor antagonists (Braszko et al., 2003; Rocha et al., 2018a). On the other hand, decreased ACE serum levels have been previously described among patients with MDD (Stelzhammer et al., 2014).

Considering the potential involvement of the RAS in the pathophysiology of BD and its potential therapeutic implications, we carried out a cross-sectional study analyzing the levels of RAS-related molecules- ACE, angiotensin-converting enzyme 2 (ACE2), angiotensin-II (Ang II), and angiotensin (1–7) [Ang-(1–7)]- among patients with BD compared to healthy controls (HC).

## Methods

### Participants

The individuals who participated in the present study were recruited from the outpatient clinics of the Department of Psychiatry of the University of Texas Health Science Center at Houston, as well as through flyers placed on the community. Our sample was composed by 30 BD patients (10 males, 20 females, age = 35.53 ± 10.59 years) and 30 matched healthy controls (10 males, 20 females, age = 34.83 ± 11.49 years). For both groups, the established inclusion criteria were: age equal or superior to 18 years, no family history of hereditary neurological disorder, no neurological or major medical condition, no current substance abuse or dependence, and a negative urine drug screening. In addition, specifically in the case of healthy controls (HC), individuals with a positive family history of mental disorders in first-degree relatives were excluded.

The diagnosis of BD among patients and the absence of psychiatric disorders in HC was established through the administration of the Structured Clinical Interview for DSM-IV Axis I Disorders-SCID (First et al., 1996). All patients met DSM-IV criteria for BD type I. At the time of their inclusion in the study, 14 BD patients were euthymic, while eight were depressed, five were manic, one was hypomanic, and two met criteria for a mixed mood state. The participants’ mood state were defined according to their answers to the SCID. Euthymic mood was defined as an absence of criteria for an acute mood episode at the time of the assessment, based on the SCID and, in addition, according to the scores of the MADRS and YMRS. The following cut offs were adopted: MADRS score of at least 7 (for the characterization of depressive mood) and YMRS of at least 12 (for mania/hypomania). Most patients (*n* = 26) were receiving one or more psychiatric medications at the time of their inclusion in the study (seven patients were on lithium, 16 on anticonvulsant mood stabilizers, 12 on antidepressants, and 17 on antipsychotics). There were no statistically significant differences between euthymic and non-euthymic patients with respect to medication status. This study was approved by the respective Institutional Review Board (HSC-MS-09-0340). Informed consent was obtained from all participants.

### Measurement of the Levels of RAS-Related Molecules

Blood samples were collected in heparin-coated collection tubes and centrifuged twice at ∼20°C for 10 min, one at 1,800 rpm and the other one at 3,000 rpm. Plasma samples were stored at −80 °C for further processing. The levels of plasma ACE (catalog # MBS727096), ACE2 (catalog # MBS723213), Ang II (catalog # MBS764273), and Ang-(1–7) (catalog # MBS084052) were assessed using Enzyme-Linked Immunosorbent Assay (ELISA), according to the manufacturer’s instructions (MyBioSource, Inc., SanDiego, CA, United States). A competitive ELISA method was used for ACE, while a sandwich ELISA procedure was used for ACE2, Ang II, and Ang-(1–7). Concentrations were measured in pg/mL (ACE, ACE2, and Ang II) and in ng/ml [Ang (1–7)]. The sensitivity of the assays was 1.0 pg/mL for ACE and ACE2, 18.75 pg/mL for Ang II, and 0.02 ng/mL for Ang (1–7). The analyses were performed blind to subject group (patients vs. controls).

### Statistical Analysis

The statistical analyses were performed using IBM SPSS statistical software (IBM SPSS, version 19, Armonk, NY) and STATA (StataCorp, 2013). The sociodemographic features of the groups were compared using exact chi-square tests (for categorical variables) and the Student’s *t*-test (for continuous variables). The levels of Angiotensin II, ACE, and other markers of RAS activity in patients and controls were compared using the Mann–Whitney *U* test. Among patients, we also looked into possible correlations (using the Spearman correlation coefficient) between the levels of RAS-related molecules and mood-rating scales, namely the Montgomery–Åsberg Depression Rating Scale (MADRS) and the Young Mania Rating Scale (YMRS).

## Results

**FIGURE 1:**
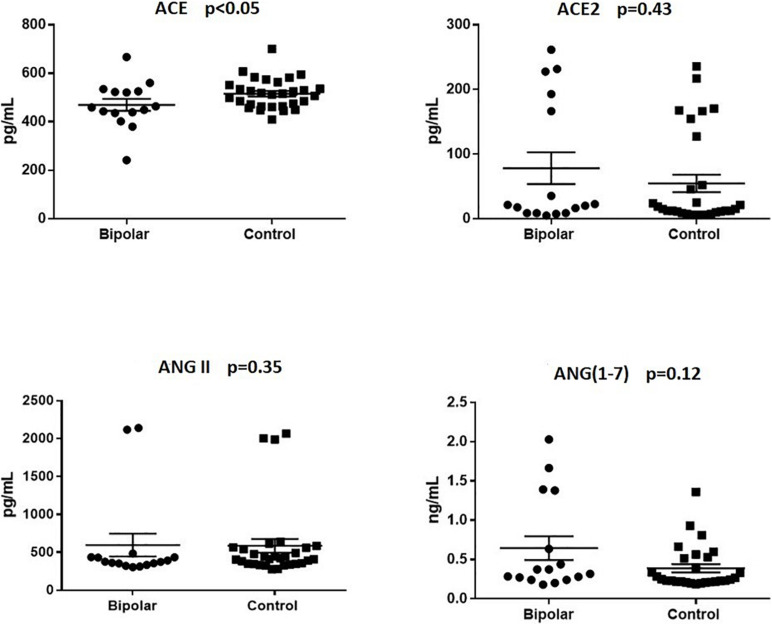
Plasma levels of renin-angiotensin system (RAS)-related molecules in non-euthymic bipolar patients and healthy controls. Non-euthymic bipolar disorder patients (*n* = 16) presented with significantly lower plasma levels of angiotensin-converting enzyme (ACE) than healthy controls (*n* = 30). Differences regarding the levels of angiotensin-converting enzyme 2 (ACE2), angiotensin-II (Ang II), and angiotensin (1–7) [Ang-(1–7)] were not statistically significant.

Both groups were matched according to age and sex, and did not differ with respect to other sociodemographic features (Table 1). No statistically significant differences between groups were observed with regards to the plasma levels of ACE, ACE2, Ang-(1–7), and Ang II (Table 1). However, a secondary analysis, including only non-euthymic BD patients and HC (Figure 1) revealed significantly lower ACE levels among patients compared to controls (median:459.00 vs. 514.10, *p* < 0.05). Differences regarding ACE2 (median: 20.73 vs. 15.91, *p* = 043), Ang II (median: 374.90 vs. 415.10, *p* = 0.35), and Ang-(1–7) [median: 0.34 vs. 0.25, *p* = 0.12) remained non-significant. Similarly, among bipolar patients, we found no statistically significant correlations between the plasma levels of the molecules of interest and the scores of the MADRS [ACE: *r* = 0.12, *p* = 0.53; ACE2: *r* = −0.01, *p* = 0.94; Ang II: *r* = −0.17, *p* = 0.37); Ang (1–7): *r* = 0.21, *p* = 0.26] or YMRS [ACE: *r* = −0.16, *p* = 0.40; ACE2: *r* = −0.14, *p* = 0.45; Ang II: *r* = 0.08, *p* = 0.69); Ang (1–7): *r* = 0.12, *p* = 0.59].

**TABLE 1:** Sociodemographic features and plasma levels of renin-angiotensin system (RAS)-related molecules in bipolar disorder patients (BD) and healthy controls.

	BD (*n* = 30)	HC (*n* = 30)	*p*
Gender (males/females)	10/20	10/20	1.00^*a*^
Age (year = mean ± SD)	35.53 ± 10.59	34.83 ± 11.49	0.81^*b*^
Education (years)	14.67 ± 1.88	15.10 ± 1.88	0.38^*b*^
Ethnicity (Hispanic/Non-Hispanic)	7/23	7/23	1.00^*a*^
SBP (mean ± SD)	123 ± 17	116 ± 19	0.18^*b*^
DBP (mean ± SD)	82 ± 12	78 ± 14	0.34^*b*^
RAS molecules (Median)			
ACE^*d*^	504.01	514.10	0.29^*c*^
ACE2^*d*^	18.82	15.91	0.71^*c*^
Ang II^*d*^	408.61	415.10	0.59^*c*^
Ang (1–7)^*e*^	0.30	0.25	0.24^*c*^

*^*a*^Chi-square test. ^*b*^Student’s *t*-test. ^*c*^ Mann-Whitney *U* test. SBP, systolic blood pressure in mmHg; DBP, diastolic blood pressure in mmHg; ACE, angiotensin-converting enzyme; ACE2, angiotensin-converting enzyme 2; Ang II, angiotensin-II; Ang-(1–7), angiotensin (1–7). ^*d*^Levels presented in pg/ml. ^*e*^Levels presented in ng/ml.*

## Discussion

Our results indicate that patients with BD in non-euthymic state had lower plasma levels of ACE when compared to healthy controls. No statistically significant differences were observed with respect to the levels of ACE2, Ang II, and Ang-(1–7). While these results may be, at least in part, interpreted as evidence against the involvement of the RAS in the pathophysiology of BD, their putative pathophysiological significance needs to be carefully discussed.

The relationship between RAS activity and mental disorders seems to be complex, with evidence suggesting a close association between RAS, inflammation, and psychiatric disorders (Saavedra, 2012; Rocha et al., 2018a; Ren et al., 2019). It has been proposed that the RAS is composed by two arms, which have opposite actions in terms of inflammatory activity and effects on the pathophysiology of mental illnesses. The first arm, composed by ACE, Angiotensin II, and Angiotensin receptor type I (AT1), displays proinflammatory actions. Increments in this pathway are hypothesized to contribute to the development of mental disorders (Rocha et al., 2018a). On the other hand, the second arm, comprised of ACE2, Ang (1–7), and Angiotensin receptor type II (AT2) seems to have anti-inflammatory effects and a putatively protective effect against the development of neuropsychiatric disorders (Mohite et al., 2018). Nevertheless, there are inconsistent patterns of findings on the possible involvement of the RAS across different psychiatric conditions.

While part of these conflicting findings may be secondary to methodological issues, including the characteristics of the subjects, it is possible that these discrepancies are related to variations in the pathophysiological factors involved in different subtypes of mood disorders. For example, some patients with mood disorders seem to have a more prominent involvement of immune factors and stress-related hyperactivation of the HPA axis. The relationship between the RAS and HPA axis is well-described and seems to be bidirectional, with the ACE contributing to the activation of the HPA axis and, in contrast, elevated cortisol levels leading to compensatory decreases in ACE levels (Stelzhammer et al., 2014). Therefore, while our finding of decreased ACE levels in BD patients may sound counterintuitive, it may suggest that, in certain groups of psychiatric patients (including patients with mood disorders and schizophrenia), abnormalities in the RAS system may occur as a downstream consequence of increases in inflammatory and HPA activity. This possibility might also explain the fact that we found significantly decreases in ACE levels among non-euthymic patients but not in euthymic patients. In other words, according to this hypothesis, decreased ACE might represent a potential state biomarker for BD but not an endophenotype/marker of vulnerability for that condition.

Our study has some methodological limitations that need to be acknowledged. First, our small sample size may, in part, explains the lack of statistically significant differences between groups regarding other RAS-related molecules. Second, our sample included only outpatients, and it is unclear whether similar findings would be applicable to a sample of patients with more severe forms of the disease. Third, most of the bipolar patients in our sample were medicated, and literature data indicates that antipsychotics are associated with increases in the CSF levels of ACE (Wahlbeck et al., 1998). While it is uncertain whether other drugs, such as lithium, other mood stabilizers, and antidepressants have similar effect, this possibility exists. Finally, we measured ACE and ACE 2 levels but not enzyme activity.

Moreover, RAS abnormalities may be involved in the pathophysiology of other mental disorders, whose symptoms might potentially overlap with the ones from BD. In spontaneous hypertensive rats, which show behavioral features correlated with hyperactivity and impulsivity and have been proposed as an animal model for attention-deficit disorder (ADHD) (Cho et al., 2014; Natsheh and Shiflett, 2018), associations between RAS and HPA activity have previously been described (Raasch et al., 2006). These finds suggest that RAS dysfunctions might play a role in the pathogenesis of ADHD. Given the commonly observed challenges involved in the differentiation between BD and ADHD due to phenotypical overlap between both conditions, one could hypothesize that some of our negative findings are related to the inadvertent inclusion of ADHD patients in our sample. While the possibility in question cannot be completely ruled out, it is unlikely that was the case, as the diagnosis of BD was confirmed by a structured psychiatric interview (SCID). Furthermore, we recruited only BD I patients, whose higher severity in terms of lifetime psychopathological features allows for a more clear distinction between BD and ADHD.

One last methodological limitation of our study is related to potential concerns about the accuracy and specificity of commercial ELISAs for the measurement of Ang II, and Ang-(1–7), which can produce results at range values distinct from those obtained through other approaches (Chappell et al., 2021). That issue may create difficulties in the comparison of our results with the ones obtained by other groups. Our findings should be considered preliminary and must be confirmed and validated by future studies utilizing different methods.

In summary, our results raise some hypothesis about the potential involvement of RAS dysfunction in the pathophysiology of BD, particularly as a putative marker of activity of that illness. The molecular mechanisms underlying this involvement, as well as its possible modulation by clinical factors such as mood state, illness severity, and treatment status among patient with BD, correspond to a promising and relatively unexplored area of study. Given the potential therapeutic implications linked to a better understanding of the role of RAS dysfunction in BD, studies with larger samples and longitudinal designs, allowing the measurement of RAS-related molecules level and activity across different mood states are of high interest.

## Data Availability Statement

The datasets for this article are not publicly available. Requests to access the datasets should be directed to MS.

## Ethics Statement

The studies involving human participants were reviewed and approved by the CPHS-UT Health Science Center at Houston. The patients/participants provided their written informed consent to participate in this study.

## Author Contributions

MS participated in the research design, data collection and analysis, data interpretation and in the drafting, revision, and approval of the final manuscript. GC participated in the data analysis and interpretation and in the drafting, revision, and approval of the final manuscript. VC and TB participated in the data collection and in the revision and the drafting, revision, and approval of the final manuscript. DR participated in the drafting, revision, and approval of the final manuscript. JS participated in the research design, data interpretation, and in the drafting, revision, and approval of the final manuscript. AT participated in the research design, data analysis and interpretation, and in the drafting, revision, and approval of the final manuscript. All authors contributed to the article and approved the submitted version.

## Conflict of Interest

The authors declare that the research was conducted in the absence of any commercial or financial relationships that could be construed as a potential conflict of interest.
